# Comprehensive bioinformatics analysis of FXR1 across pan-cancer: Unraveling its diagnostic, prognostic, and immunological significance

**DOI:** 10.1097/MD.0000000000036456

**Published:** 2023-12-01

**Authors:** Keyuan Xiao, Ihsan Ullah, Fan Yang, Jiao Wang, Chunxia Hou, Yuqiang Liu, Xinghua Li

**Affiliations:** a Changzhi People’s Hospital Affiliated to Changzhi Medical College, Changzhi, China; b National Chinmedomics Research Center, Heilongjiang University of Chinese Medicine, Harbin, China.

**Keywords:** FXR1, immune cell infiltration, mutation, pan-cancer, survival analysis

## Abstract

Fragile X-related protein 1 (FXR1) is an RNA-binding protein that belongs to the fragile X-related (FXR) family. Studies have shown that FXR1 plays an important role in cancer cell proliferation, invasion and migration and is differentially expressed in cancers. This study aimed to gain a comprehensive and systematic understanding of the analysis of FXR1’s role in cancers. This would lead to a better understanding of how it contributes to the development and progression of various malignancies. this study conducted through The Cancer Genome Atlas (TCGA), GTEx, cBioPortal, TISIDB, GEPIA2 and HPA databases to investigated FXR1’s role in cancers. For data analysis, various software platforms and web platforms were used, such as R, Cytoscape, hiplot plateform. A significant difference in FXR1 expression was observed across molecular and immune subtypes and across types of cancer. FXR1 expression correlates with disease-specific survival (DSS), and overall survival (OS) in several cancer pathways, further in progression-free interval (PFI) in most cancers. Additionally, FXR1 showed a correlation with genetic markers of immunomodulators in different cancer types. Our study provides insights into the role of FXR1 in promoting, inhibiting, and treating diverse cancers. FXR1 has the potential to serve as a diagnostic and prognostic biomarker for cancer, with therapeutic value in immune-based, targeted, or cytotoxic treatments. Further clinical validation and exploration of FXR1 in cancer treatment is necessary.

## 1. Introduction

Cancer is the most deadly disease and seriously affects the quality of life, endangers human health worldwide, and imposes a heavy economic burden on society. The aim of pan-cancer analysis is to use bioinformatics tools to transversally analyze the role of target genes in different tumors.^[1,2]^ Given the complex nature of tumorigenesis, it is crucial to conduct pan-cancer expression analysis of genes of interest to evaluate their clinical relevance and potential molecular mechanisms. Fragile X-related protein 1 (FXR1) is an RNA-binding protein that belongs to the fragile X-related (FXR) family. It is situated on chromosome 3q26.3. FXR1 is primarily found in the nervous system and muscle tissue, playing an important role in normal muscle growth and myogenesis.^[3–5]^ Recent studies have revealed that FXR1 plays a crucial role in the advancement of tumors and is linked to unfavorable outcomes in a range of human cancer types, such as breast, ovarian, head and neck, and non-small cell lung cancers.^[6–8]^ In non-small cell lung cancer, FXR1 is overexpressed in tissues and cells. The silencing of FXR1 leads to decreased cell growth and increased cell apoptosis ratio by destabilizing ECT2 mRNA.^[3]^ Furthermore, FXR1 expression is elevated in squamous cell carcinoma of the head and neck. FXR1 degradation mediated by Fbxo4 inhibits tumorigenesis in head and neck squamous cell carcinoma, while FXR1 regulates Fbxo4 expression by inhibiting protein translation in feedback.^[9]^ FXR1 expression is elevated in nephroblastoma, and the expression level of FXR1 in nephroblastoma patients is 10 times that of the normal population. Inhibition of FXR1 may induce terminal differentiation of nephroblastoma. However, FXR expression level is significantly decreased in squamous cell carcinoma tissues. Studies have shown that overexpression of FXR can induce apoptosis of early and advanced cancer cells, promote G1 arrest, and inhibit cervical cancer through up-regulation of SHP, MDM2 and p53.^[10,11]^

Despite the growing body of research on the connection between FXR1 and cancer, there is a scarcity of comprehensive pan-cancer analyses focusing on FXR1. In this study, we investigated the expression difference of FXR1 in different tumors, its influence on tumor microenvironment and tumor prognosis, and the influence of FXR1 gene expression on tumor proliferation. The findings demonstrate that FXR1 could play a significant prognostic and diagnostic role in a wide range of cancers and its multifaceted role in cancer regulation. Additionally, we discovered previously unreported mechanisms and pathways through which FXR1 influences cancer progression. Moreover, previous studies have seldom explored the connection between FXR and tumor immunomodulation. This new finding could enhance our comprehension of FXR1’s involvement in cancer immunomodulation and its potential implications for immunotherapy.

## 2. Materials and methods

### 2.1. Gene expression analysis

We retrieved expression profile data and clinical information for a cohort of 33 tumors from TCGA database (https://cancergenome.nih.gov) and GTEx database (https://gtexportal.org/), and then the Human normal and tumor tissue immunohistochemical (IHC) images were sourced from HPA database (https://www.proteinatlas.org/).Gene expression levels are represented as log2 transcript-per-million values.

### 2.2. Receiver operator characteristic (ROC) analysis

We employed ROC analysis to evaluate FXR1’s diagnostic utility across 33 cancer types. ROC curves were generated using R software’s “Proc” (v1.17.0.1) and visualized with “ggplot2.” Additionally, we computed the Area under Curve (AUC). The higher the AUC, the greater the diagnostic performance. For example, AUC values between 0.5 and 0.7 indicate low accuracy, 0.7 and 0.9 indicate good accuracy, while values > 0.9 indicate high accuracy.

### 2.3. Survival prognosis analysis

In the context of survival analysis in pan-cancer, clinical phenotypic and survival data were meticulously gathered from the TCGA database. Subsequently, Kaplan–Meier (K-M) analysis, accompanied by a log-rank test, was executed utilizing the “survival” package. The primary objective was to discern disparities in DSS (disease-specific survival); OS (overall survival); and PFI (progress-free interval), rates between groups characterized by low and high FXR1 gene expression levels. Cox regression analysis was performed to ascertain statistical significance, which yielded a corresponding *P* value. Based on a calculation of hazard ratios (HR), 95% confidence intervals, and *P* values for survival curves, and through visualization techniques employing the “survminer” and “ggplot2” packages, a forest map was drawn in ultimately.

### 2.4. FXR1 expression in the landscape of cancer molecular and immune subtypes

We investigated the correlation between FXR1 expression and immune or molecular subtypes across 33 cancer types using the “subtype” module of the TISIDB database (http://cis.hku.hk/TISIDB/), which integrates diverse data modalities to evaluate the interplay between the immune system, and cancer. FXR1 mRNA expression was assessed within distinct immune subtypes and molecular subtypes.

### 2.5. Genetic alteration analysis

To investigate genetic alterations in FXR1, we employed the cBioPortal (https://www.cbioportal.org/). We analyzed somatic mutation frequencies and genomic alterations of FXR1 across various cancer types based on the “cancer types summary and mutations” and “mRNA vs study” modules. Specific mutation sites were extracted from the mutation’s module.

### 2.6. FXR1-related gene enrichment analysis

The “Similar Gene Detection” module of the GEPIA2 database was used to obtain the top 100 FXR1-associated targeted genes based on all TCGA tumor and normal tissue datasets. To analyze protein-protein interactions (PPI) involving FXR1, we leveraged the STRING database (https://string-db.org/) to compile potential interaction data. Network analysis of relevant genes was carried out using a confidence score threshold of 0.7, and we further imported this data into Cytoscape software for visualization and further exploration. Key modules with “top 10 nodes” were identified using Cytoscape’s cytoHubba plugins, and the top 10 hub genes, ranked by MCC, were determined. GO (Gene ontology) and KEGG (Koto Encyclopedia of Genes and Genomes) pathway enrichment analysis was conducted on the Hiplot platform (https://hiplot.com.cn/). GO enrichment analysis provided results for biological processes, chemical functions, and cellular components. In order to visualize the 10 top entries, bubble plots were generated based on enrichment analysis with a *P* value < .05 for each entry.

### 2.7. Gene set enrichment analysis (GSEA)

For GSEA, the “cluster Profiler” package was used to compare the biological pathway differences between high- and low-FXR1 groups.

### 2.8. Immunogenomic analyses

**Figure 1.**
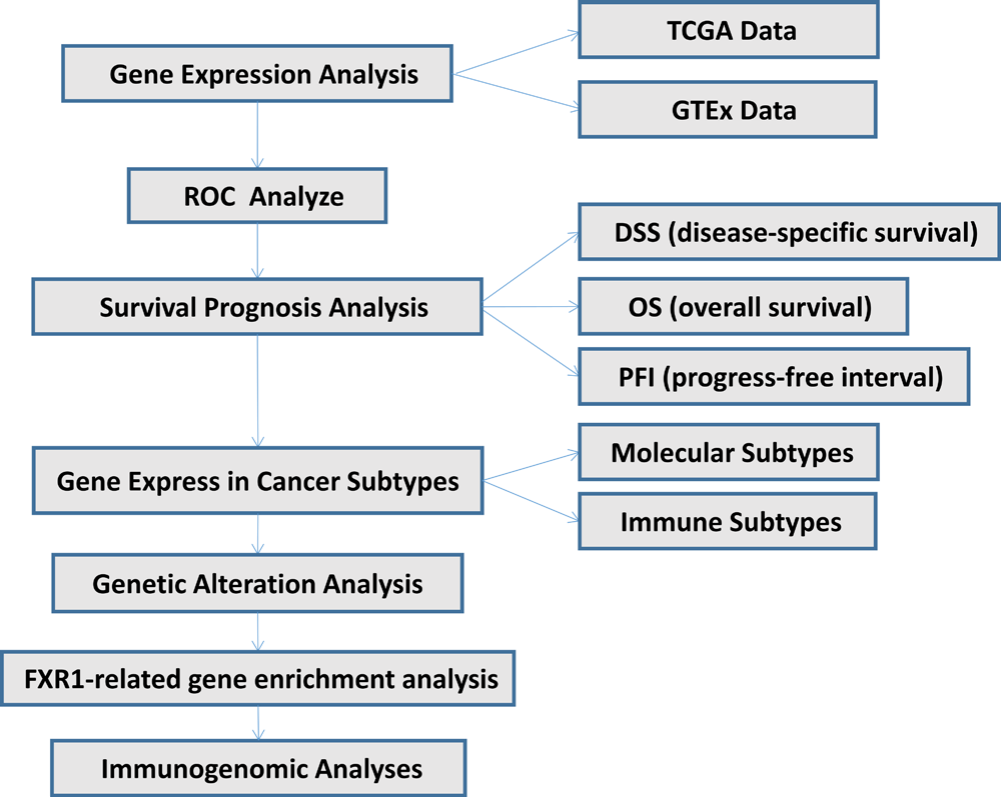
Flow chart of the study.

In order to investigate the relationship between FXR1 expression and various immune-related factors in pan-cancer, we used the “GSVA” package with the “ssGSEA” algorithm. Furthermore, In this comprehensive study, we assessed various factors, including tumor-infiltrating lymphocytes, immunostimulatory and immunoinhibitory molecules, MHC (major histocompatibility complex) molecules, chemokine receptors, and chemokines, across 33 distinct cancer types. The Spearman correlation coefficient was employed to quantify the associations, with statistical significance established at *P* values below < .05. The resultant correlations were visually represented as heat maps to facilitate interpretation, employing the “ggplot2” package. Figure 1 shows our study flow chart.

### 2.9. Statistical analysis

An LOG2 transformation was used to normalize all gene expression data. Statistically significant results were obtained by contrasting cancer tissues and non-tumor normal tissues through the use of 2 groups of *t* tests; *P* < .05 indicates that the results are significant. Throughout this study, survival analysis was conducted using log-rank tests, KM curves, and Cox proportional risk regression models. An analysis of the relationship between the 2 variables was conducted using Spearman’s test. A significant difference was determined by *P* < .05. R software (version 4.2.1) was used to perform the statistical analysis in this study.

## 3. Results

### 3.1. The expression level of FXR1 in pan-cancer

**Figure 2.**
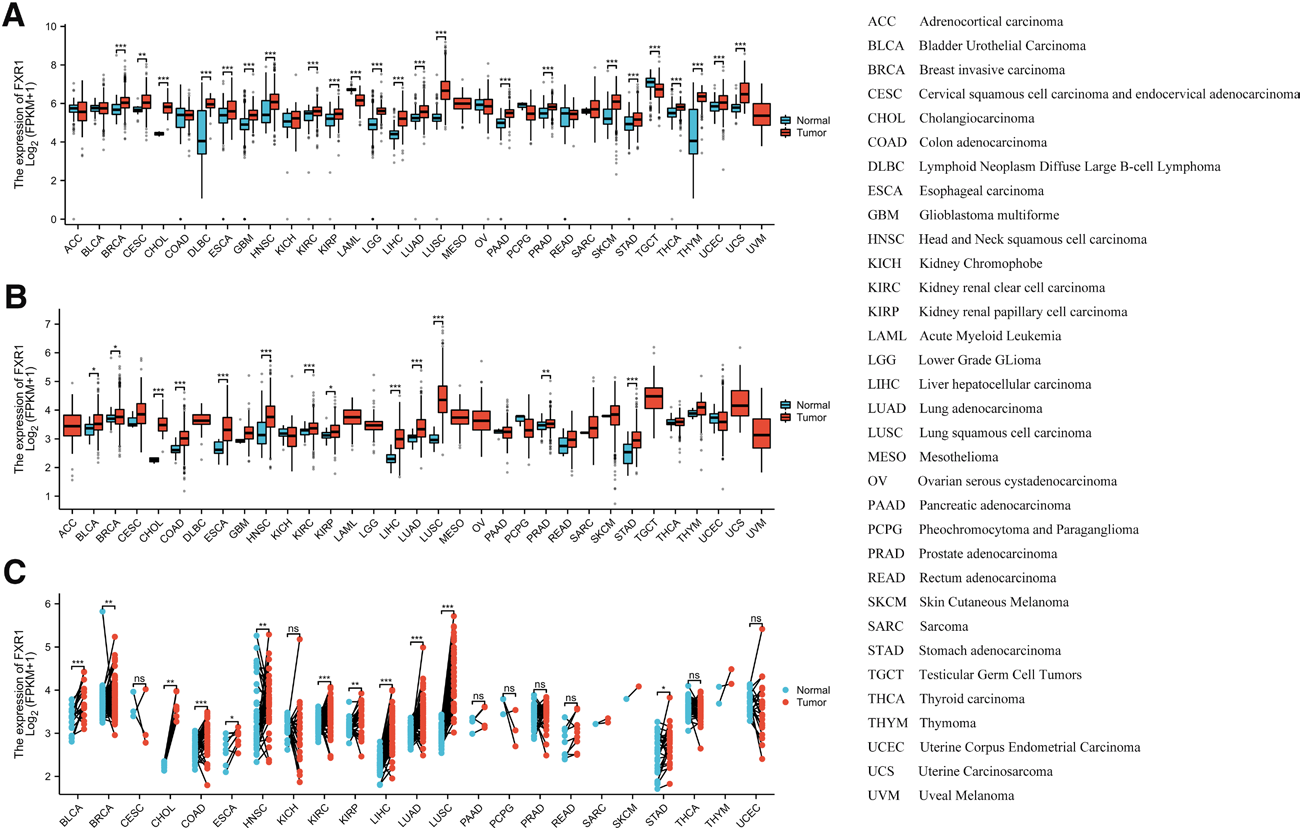
The pan-cancer FXR1 mRNA expression. (A) The mRNA levels of FXR1 within a cohort of 33 tumors sourced from the TCGA GTEx database. (B) Analysis of FXR1 mRNA expression across 33 tumors in the TCGA database. (C) FXR1 expression levels in 23 tumor pairs from TCGA database exhibit significant differences (*P < .05; **P < .01; ***P < .001). FXR1 = fragile X related protein 1, TCGA = The Cancer Genome Atlas.

**Figure 3.**
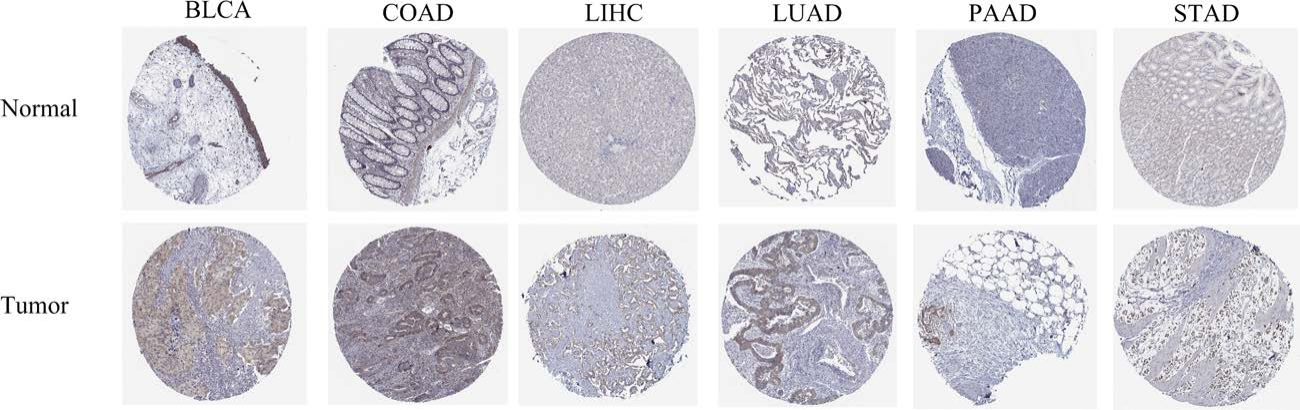
The immunohistochemical (IHC) images showing FXR1 expression in both normal and tumor tissues obtained from the HPA. FXR1 = fragile X related protein 1.

In order to clarify the expression of FXR1 in pan-cancer, the FXR1 mRNA-expression levels in the TCGA and GTEx databases were analyzed. The findings indicated significant variations in FXR1 expression (Fig. 2A), across most tumor types. Analysis of a comprehensive dataset comprising 18,102 samples sourced from TCGA database demonstrated that compared with normal samples, a notably high FXR1 mRNA expression was found in BRCA, CHOL, DLBC, ESCA, GBM, HNSC, KIRC, KIRP, LGG, LIHC, LUAD, LUSC, STAD, SKCM, PRAD, PAAD, THYM, UCEC, UCS (*P* < .001), CESC (*P* < .01), and low FXR1 mRNA expression in LAML, TGCT (*P* < .001). Due to the lack of sufficient normal samples, MESO and UVM could not be analyzed (Fig. 2A). Figure 2B depicts an analysis comprising 11,123 samples sourced from GTEx data. As compared to paracancerous tissue, The expression of FXR1 mRNA was upregulated in COAD, ESCA, HNSC, LUAD, KIRC, LIHC, STAD, LUSC (*P* < .001), PRAD (*P* < .01), BLCA, BRCA, KIRP (*P* < .05) (Fig. 2B). Furthermore, we analyzed FXR1 mRNA expression in 23 different tumor types with paired samples from TCGA. Notably, FXR1 mRNA was significantly upregulated in BLCA, BRCA, CHOL, COAD, KIRC, LIHC, LUAD, LUSC (*P* < .001), HNSC (*P* < .01), and ESCA, STAD (*P* < .05) (Fig. 2C). Additionally, We extracted IHC images depicting both tumor and normal tissues across BLCA, COAD, LIHC, LUAD, PAAD, and STAD, as illustrated in Figure 3.

### 3.2. ROC analysis of FXR1 in pan-cancer

**Figure 4.**
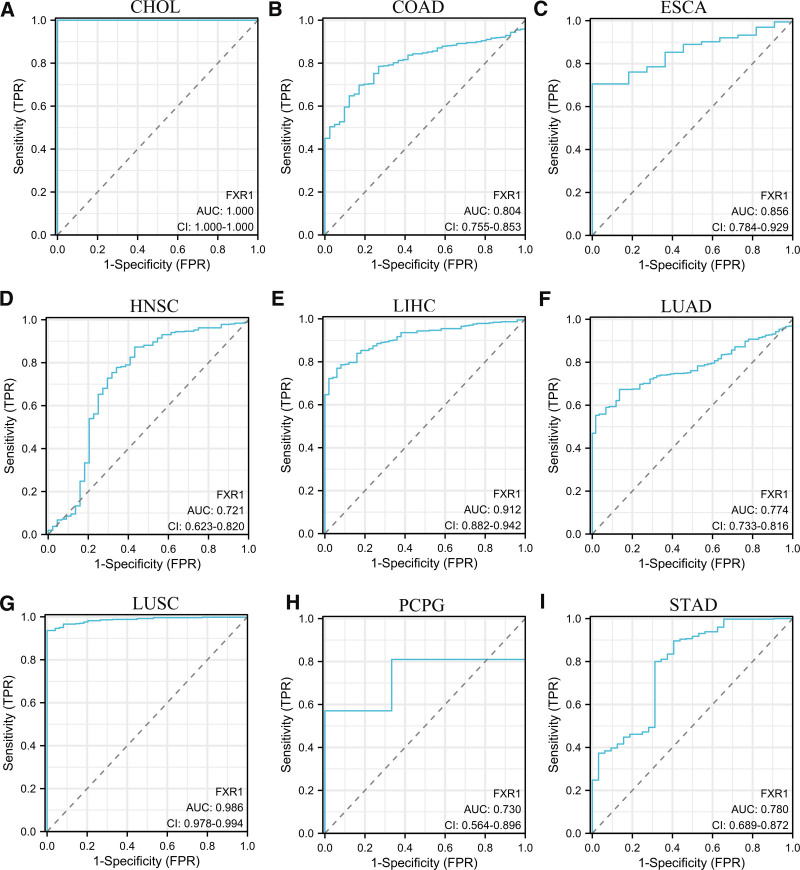
The ROC curve showing the discriminative performance of FXR1 across 9 distinct cancer types, demonstrating an AUC (area under the curve) value exceeding > 0.9. (A) CHOL, (B) COAD, (C) ESCA, (D) HNSC, (E) LIHC, (F) LUAD, (G) LUSC, (H) PCPG, (I) STAD. CHOL = cholangiocarcinoma, COAD = colon adenocarcinoma, ESCA = esophageal carcinoma, FXR1 = fragile X related protein 1, HNSC = head and neck squamous cell carcinoma, LIHC = liver hepatocellular carcinoma, LUAD = lung adenocarcinoma, LUSC = lung squamous cell carcinoma, PCPG = pheochromocytoma and paraganglioma, STAD = stomach adenocarcinoma.

The diagnostic efficacy of FXR1 across diverse tumor types was assessed using ROC curves in a cohort of 33 different cancers. Its AUC surpassed the threshold of 0.7 in 9 cancer types, including CHOL (1.000), COAD (0.804), ESCA (0.856), HNSC (0.721), LIHC (0.912), LUAD (0.774), LUSC (0.986), PCPG (0.730), and STAD (AUC = 0.780), as shown in Figure 4A–I. Notably, 3 cancer types exhibited AUC values exceeding 0.9. Consequently, these findings underscore the robust diagnostic potential of FXR1 across various cancer types.

### 3.3. Prognostic value of FXR1 in pan-cancer

**Figure 5.**
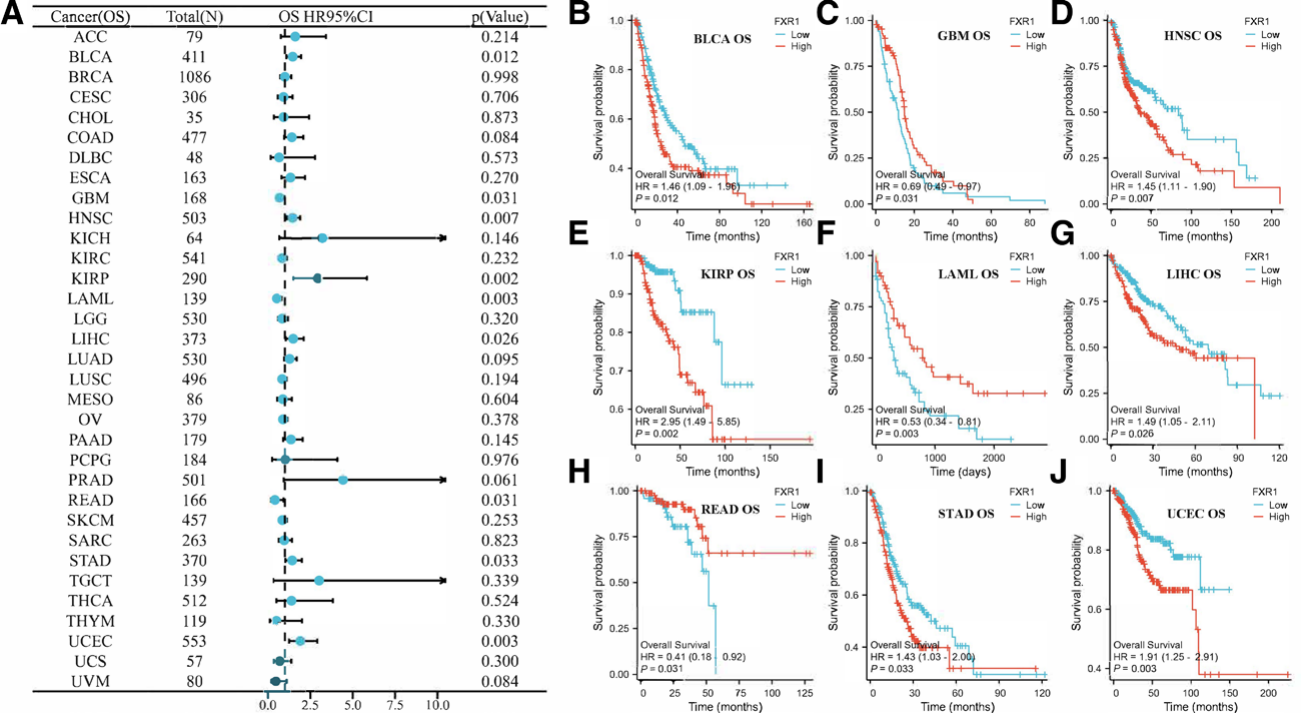
The relationship between FXR1 expression and overall survival (OS) across a spectrum of 33 different cancers, as depicted in a forest plot. Subsequently, sections (B–E) delve into the specific impact of FXR1 expression on OS in BLCA, GBM, HNSC, KIRP, LAML, LIHC, READ, STAD, and UCEC, respectively. BLCA = bladder urothelial carcinoma, FXR1 = fragile X related protein 1, GBM = glioblastoma multiforme, HNSC = head and neck squamous cell carcinoma, KIRP = kidney renal papillary cell carcinoma, LAML = acute myeloid leukemia, LIHC = liver hepatocellular carcinoma, READ = rectum adenocarcinoma, STAD = stomach adenocarcinoma, UCEC = Uterine Corpus Endometrial Carcinoma.

**Figure 6.**
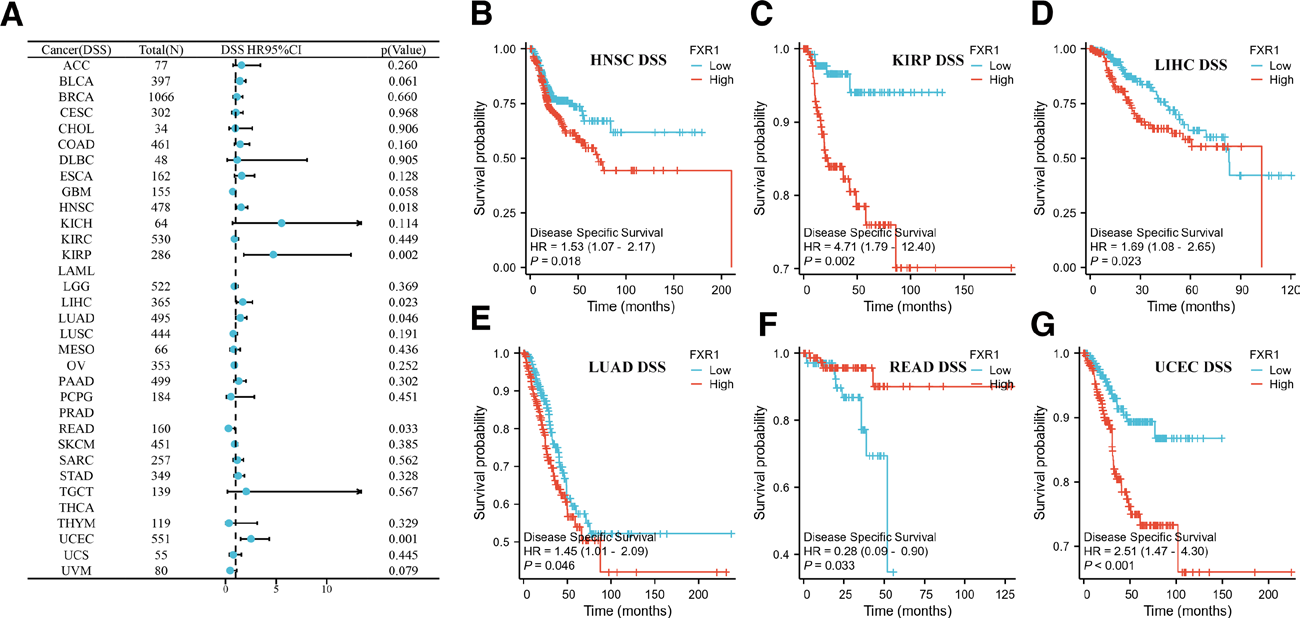
The correlation between FXR1 expression and Disease-Specific Survival (DSS) across various cancers. (A) A forest plot displays the impact of FXR1 expression on DSS in 33 different cancer types. (B–G) The influence of FXR1 expression on DSS is examined in HNSC, KIRP, LIHC, LUAD, READ, and UCEC, respectively. DLBC = lymphoid neoplasm diffuse large B-cell lymphoma, FXR1 = fragile X related protein 1, HNSC = head and neck squamous cell carcinoma, KIRP = kidney renal papillary cell carcinoma, LIHC = liver hepatocellular carcinoma, LUAD = lung adenocarcinoma, READ = rectum adenocarcinoma, UCEC = Uterine Corpus Endometrial Carcinoma.

**Figure 7.**
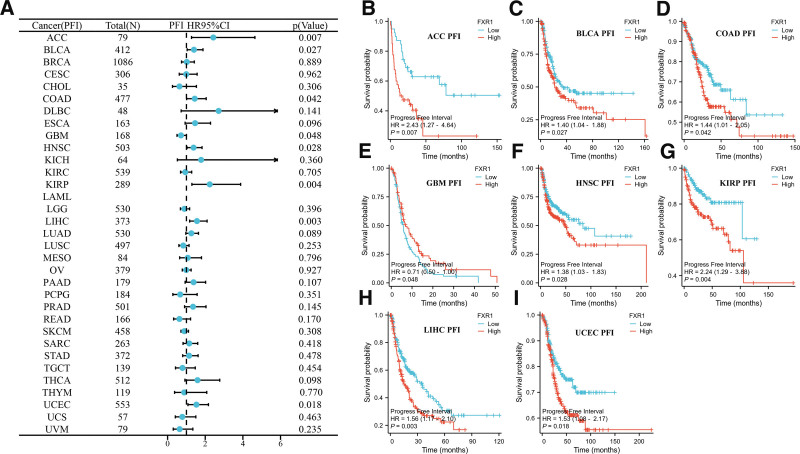
The connection between FXR1 gene expression and the Pan-Cancer Progression-Free Interval (PFI) in a multi-cancer context. (A) A forest plot was used to demonstrate how FXR1 expression influenced progression-free interval (PFI) across 33 different cancer types. (B–I) The impact of FXR1 expression on PFI in ACC; BLCA; COAD; GBM; HNSC; KIRP; LIHC; and UCEC; respectively. FXR1 = fragile X related protein 1. ACC = adrenocortical carcinoma, BLCA = bladder urothelial carcinoma, COAD = colon adenocarcinoma, GBM = glioblastoma multiforme, HNSC = head and neck squamous cell carcinoma, KIRP = kidney renal papillary cell carcinoma, LIHC = liver hepatocellular carcinoma, UCEC = Uterine Corpus Endometrial Carcinoma.

We conducted Kaplan–Meier survival analysis to evaluate FXR1’s prognostic relevance. Our findings indicate that FXR1 expression significantly impacts overall survival (OS) in 9 cancers, as shown in Figure 5. Notably, elevated FXR1 expression is associated with shorter OS in BLCA, HNSC, KIRP, LIHC, STAD, and UCEC, whereas it is linked to longer OS in GBM, LAML, and READ. As shown in Figure 6A, a correlation analysis was performed to examine FXR1 and DSS expression levels. Furthermore, FXR1 expression is correlated with disease-specific survival (DSS) in HNSC, KIRP, LIHC, LUAD, READ, and UCEC (Fig. 6B–G), with high FXR1 expression predicting worse DSS in most cases, except in READ. Regarding the relationship between FXR1 expression and Progression-Free Interval (PFI), the forest plot depicted a connection between higher FXR1 expression and PFI in COAD, BLCA, ACC, GBM, HNSC, KIRP, LIHC, and UCEC (Fig. 7A). K-M analysis showed that most tumors with high FXR1 expression had poor PFI, but the results were reversed in GBM (Fig. 7B–I).

### 3.4. Molecular and immune subtypes and expression of FXR1 in pan-cancer

**Figure 8.**
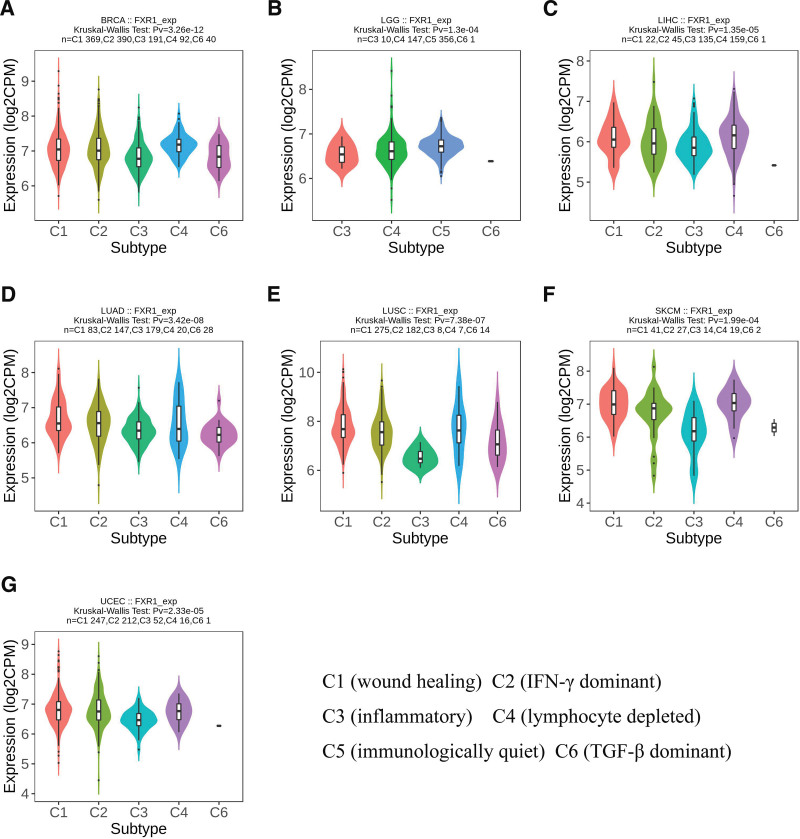
The correlations between FXR1 expression and immune subtypes in 7 cancers. (A) BRCA, (B) LGG, (C) LIHC, (D) LUAD, (E) LUSC, (F) SKCM, (G) UCEC. C1: wound healing, C2: IFN-g dominant, C3: inflammatory, C4: lymphocyte deplete, C5: immunologically quiet, and C6: TGF-β dominant. BRCA = breast invasive carcinoma, FXR1 = fragile X related protein 1, LGG = brain lower grade glioma, LIHC = liver hepatocellular carcinoma, LUAD = lung adenocarcinoma, LUSC = lung squamous cell carcinoma, SKCM = skin cutaneous melanoma, UCEC = Uterine Corpus Endometrial Carcinoma.

**Figure 9.**
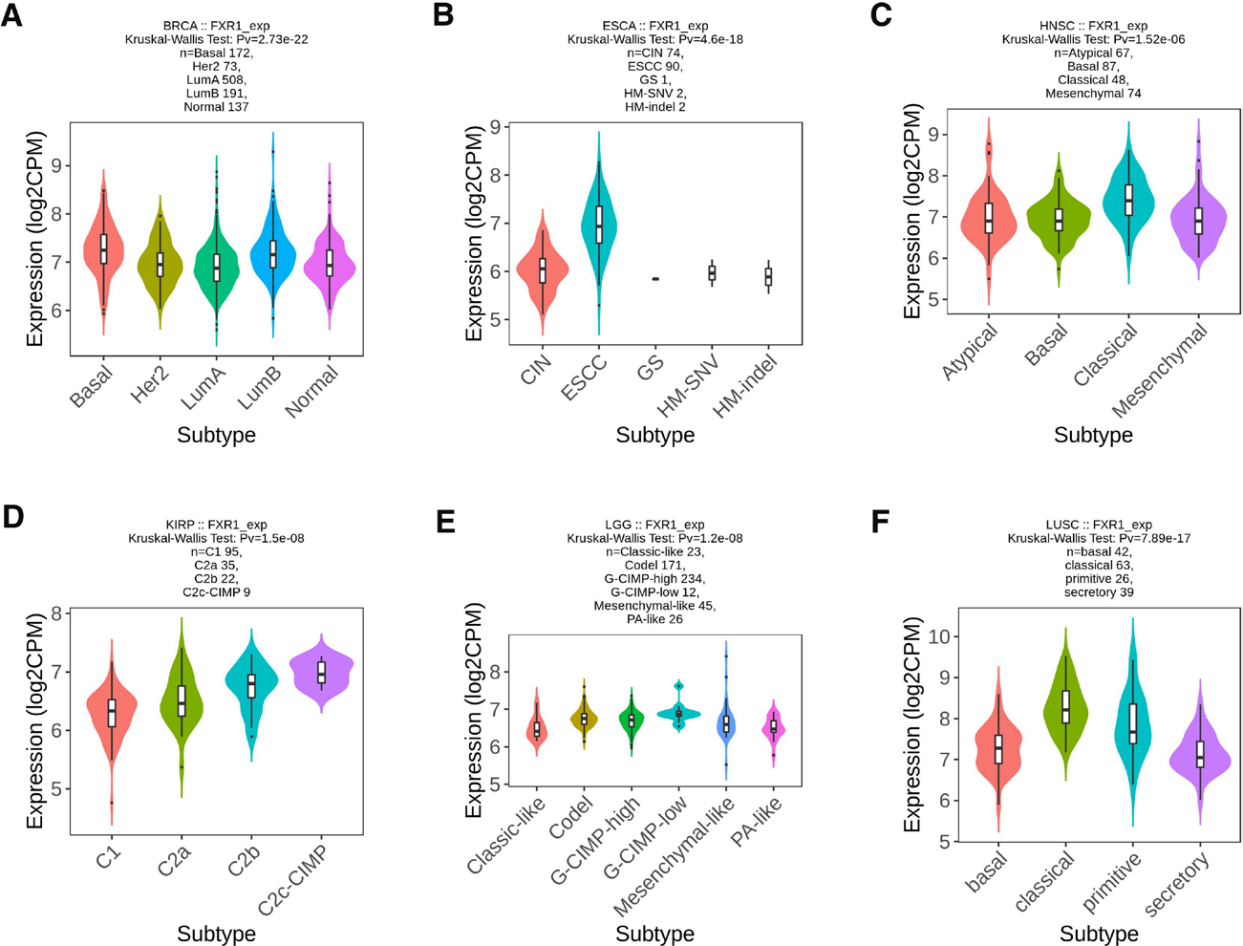
The correlations between FXR1 expression and molecular subtypes across 6 distinct cancers: (A) BRCA, (B) ESCA, (C) HNSC, (D) KIRP, (E) LGG, and (F) LUSC. BRCA = breast invasive carcinoma, ESCA = esophageal carcinoma, FXR1 = fragile X related protein 1, HNSC = head and neck squamous cell carcinoma, KIRP = kidney renal papillary cell carcinoma, LGG = brain lower grade glioma, LUSC = lung squamous cell carcinoma.

We conducted an analysis of FXR1 expression across 33 different cancers, categorizing them into both immune and molecular subtypes. Our findings reveal significant variations in FXR1 expression among immune subtypes in 7 cancers, namely BRCA (comprising 5 subtypes) as depicted in Figures 8A, LGG (with 4 subtypes) as shown in Figures 8B, LIHC (featuring 5 subtypes) as illustrated in Figures 8C, LUAD (comprising 5 subtypes) as presented in Figures 8D, LUSC (with 5 subtypes) as detailed in Figures 8E, SKCM (featuring 5 subtypes) as visualized in Figures 8F, and UCEC (with 5 subtypes) as represented in Figures 8G. Furthermore, within the molecular subtypes, FXR1 exhibited significant differences in expression across 6 cancer types: BRCA, ESCA, HNSC, KIRP, LGG, and LUSC, as illustrated in Figure 9A–F.

### 3.5. Genetic alteration differences of FXR1 in cancers

**Figure 10.**
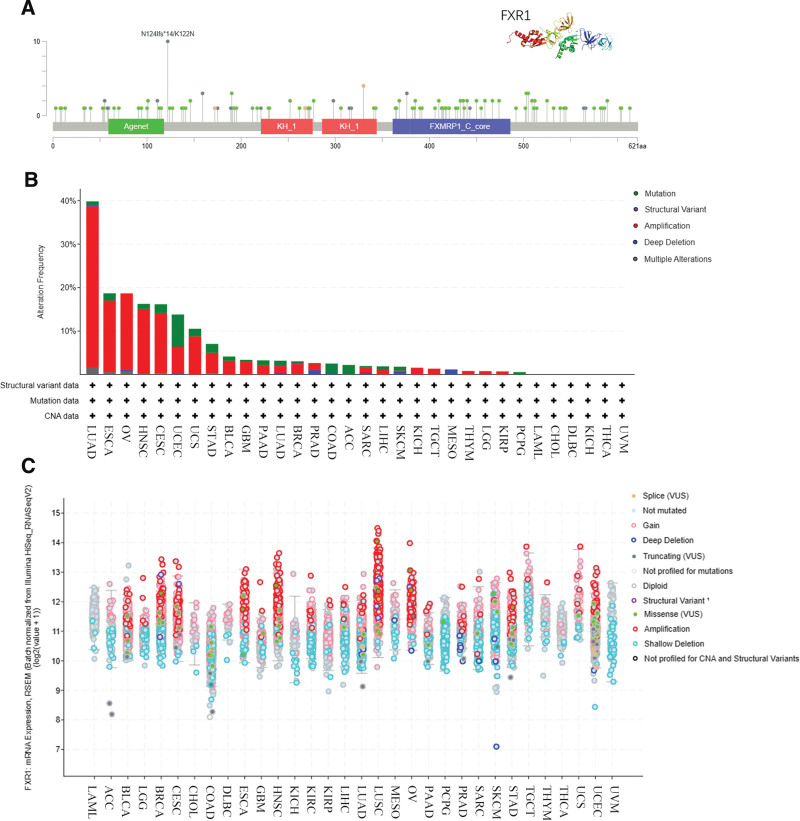
FXR1 gene mutations in 32 cancers. (A) An illustration of the mutations occurring across FXR1 protein domains. (B) An analysis of 32 cancer studies using TCGA Pan Cancer Atlas data shows FXR1 mutations in 32 studies. (C) Numbers and types of mutations in 32 cancers based on FXR1. FXR1 = fragile X related protein 1, TCGA = The Cancer Genome Atlas.

The genomic alterations affecting the expression of FXR1 in various cancer types were assessed by leveraging the cBioPortal online tool. Our analysis encompassed all 32 studies within TCGA Pan-Cancer Atlas, incorporating a total of 10,967 samples. Within the coding region spanning amino acids 0 to 621, we identified 210 distinct mutation sites. These mutations comprised 161 missense mutations, 32 truncating mutations, 6 splice-site mutations, and 11 fusion events. Notably, the K122N mutation emerged as the most prevalent among them (as depicted in Fig. 10A). Predominant mutation types observed included missense mutations, gene amplifications, and deep deletions. FXR1 mutations exhibited the highest prevalence in specific cancer types, notably LUAD, ESCA, OV, HNSC, CESC, UCEC, UCS, and STAD (refer to Fig. 10B). Amplification of FXR1 mRNA expression was prevalent in all of the 32 cancers examined, with the exceptions being LAML, ACC, CHOL, COAD, DLBC, KICH, MESO, PCPG, THCA, and UVM (as illustrated in Fig. 10C).

### 3.6. FXR1-related gene enrichment analysis

**Figure 11.**
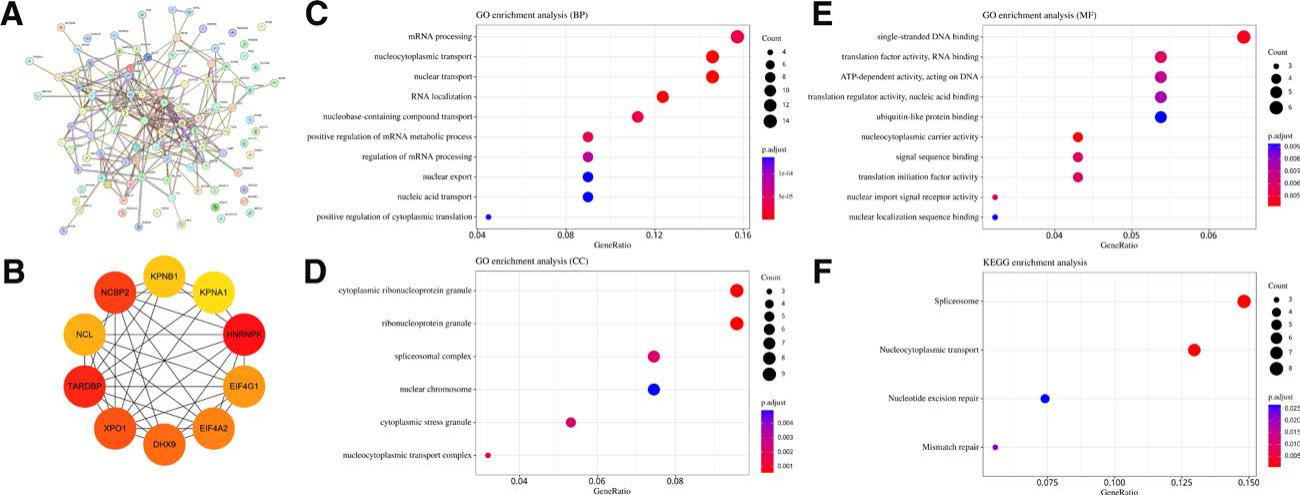
FXR1-related gene enrichment analysis. (A) the PPI network of FXR1. (B) the PPI network of top ten hub genes. (C–F) GO enrichment analysis for 100 genes related to FXR1, spanning categories of biological processes (BP), molecular functions (MF), and cellular components (CC). (F) KEGG pathways for100 genes related to FXR1. FXR1 = fragile X related protein 1.

For further investigation of the molecular mechanism involved in FXR1 tumorigenesis, pathway enrichment analyses were conducted on FXR1 expression-correlated genes. By employing the GEPIA2 database, we obtained a total of 100 FXR1 expression-correlated genes. The PPI results of 100 FXR1 expression-correlated genes were obtained through the STRING database (Fig. 11A). The top 10 hub genes were identified, including TARDBP, HNRNPK, NCBP2, XPO2, DHX9, EIF4A2, EIF4G1, NCL, KPNB1, and KPNA1 (Fig. 11B). Gene Ontology analysis revealed their involvement in mRNA processing, nucleocytoplasmic transport, nuclear transport, and other molecular processes (as shown in Fig. 11C), while they were associated with cytoplasmic ribonucleoprotein granules, ribonucleoprotein granules as well as other components of the cell (Fig. 11D). It involves various molecular functions, including but not limited to “single-stranded DNA binding,” “translation factor activity coupled with RNA binding,” and “ATP-dependent actions on DNA,” as represented in (Fig. 11E). Furthermore, KEGG pathway analysis indicated potential links to Spliceosome, Nucleocytoplasmic transport, Nucleotide excision repair, and Mismatch repair, as illustrated in (Fig. 11F).

### 3.7. GSEA analysis

**Figure 12.**
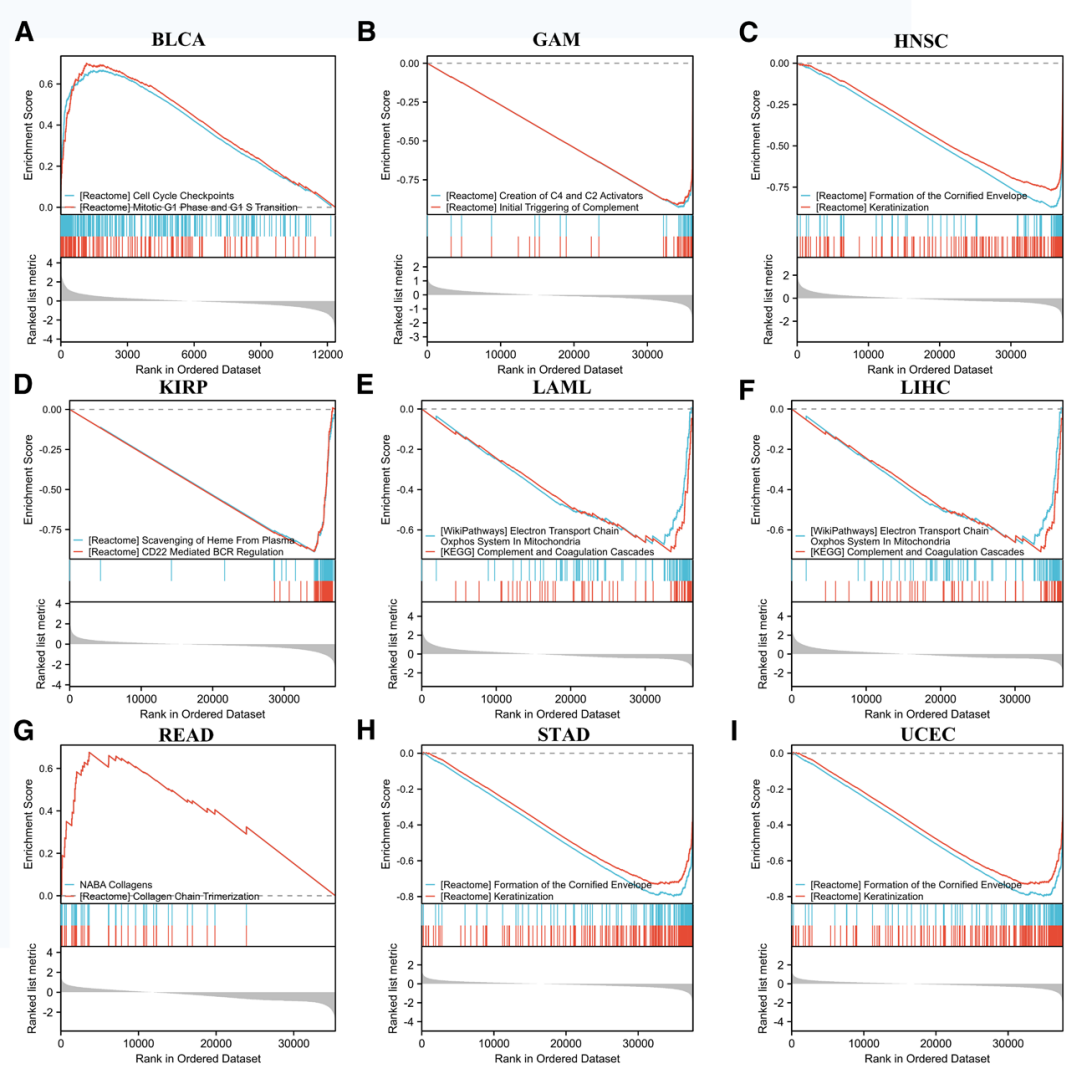
GSEA based on the differentially expression analysis in BLCA, GAM, HNSC, KIRP, LAML, LIHC, READ, STAD and UCEC, respectively. BLCA = bladder urothelial carcinoma, GBM = glioblastoma multiforme, HNSC = head and neck squamous cell carcinoma, KIRP = kidney renal papillary cell carcinoma, LAML = acute myeloid leukemia, LIHC = liver hepatocellular carcinoma, READ = rectum adenocarcinoma, STAD = stomach adenocarcinoma, UCEC = Uterine Corpus Endometrial Carcinoma.

A differential expression analysis of FXR1 was performed in the cancers where FXR1 can negatively affect prognosis in order to determine the biological value of FXR1 expression in various tumor tissues. The outcomes of the analysis indicate a predominant association of FXR1 with cellular cycle checkpoints, the electron transport chain within mitochondria (Oxidative Phosphorylation System), as well as processes related to the complement and coagulation cascades, and the formation of the cornified envelope (Fig. 12).

### 3.8. Immunogenomic analyses

**Figure 13.**
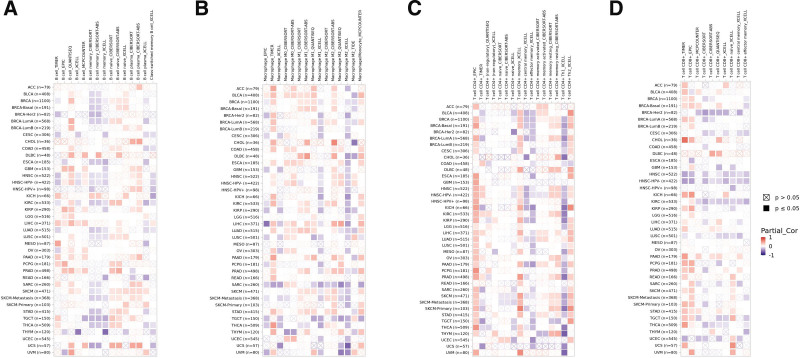
The correlation of FXR1 expression and immune cell infiltration. (A–D) Heat maps of correlations between FXR1 expression and B cells, macrophages, T cell CD4^+^, and T cell CD8^+^ in TIMER2 database, respectively. FXR1 = fragile X related protein 1.

The immune microenvironment strongly influences tumor development. This study examined the relationship between FXR1 and the immune microenvironment in pan-cancer using the GEPIA2 database. The correlation between FXR1 expression and B cells, macrophages, T CD4 + cells, and T CD8 + cells is shown in Figure 13A–D, respectively.

**Figure 14.**
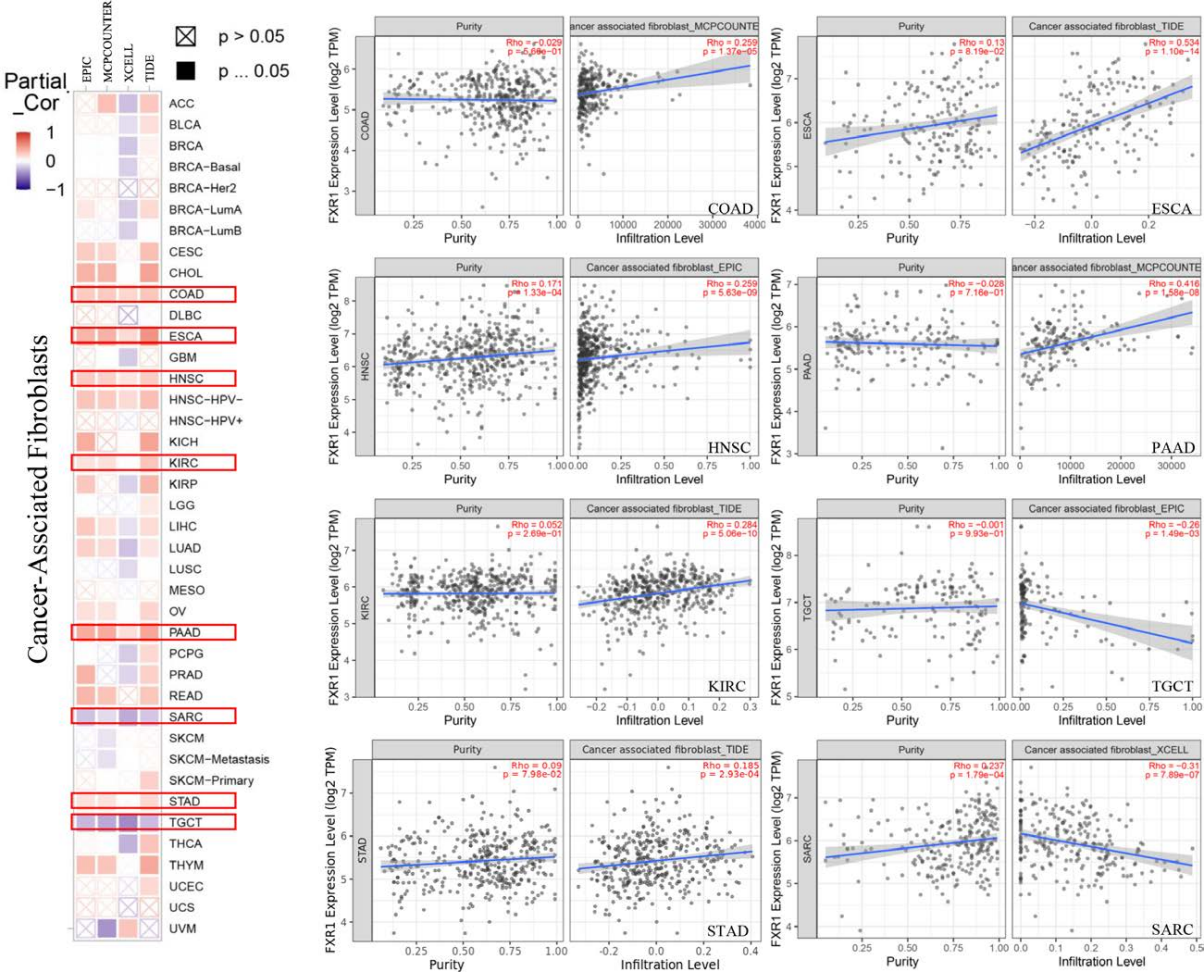
The correlation between FXR1 expression and immune infiltration of cancer-associated fibroblasts in Different algorithms across all types of cancer in TCGA. FXR1 = fragile X related protein 1, TCGA = The Cancer Genome Atlas.

Moreover, we observed a statistically positive correlation between FXR1 expression and the estimated infiltration value of cancer-associated fibroblasts in COAD, ESCA, HNSC, KIRC, PAAD and STAD but noted a negative correlation for SARC and TGCT. The scatterplot data of the above tumors produced using one algorithm are presented in Figure 14, for example, the FXR1 expression level in SARC is negatively correlated with the infiltration level of cancer-associated fibroblasts based on the XCELL algorithm.

## 4. Discussion

The role of FXR1 in cancer has been studied for its potential role in regulating tumor progression, leading to genetic overexpression in certain cancers such as human lung squamous cell carcinoma, non-small cell lung cancer, and colorectal cancer.^[3,12,13]^ However, no study has yet analyzed the potential roles of FXR1 from a pan-cancer perspective. Our verification of FXR1 overexpression in numerous types of tumors, with some results corroborated by previous studies, supports this perspective.^[6,14–16]^ The prognostic implications and potential impact on tumorigenic processes of FXR1 expression levels in various human cancers remain unexplored and necessitate additional research. To our knowledge, this study represents the inaugural comprehensive examination of FXR1’s expression and biological functionality from a pan-cancer viewpoint.

Our pan-cancer analysis revealed a significant upsurge in FXR1 expression across 25 cancer types, including BRCA, CESE, and DLBC. Immunohistochemical staining results, used to analyze FXR1 protein levels, corroborated these findings. The ROC’s AUC surpassed 0.7 in 9 cancers, indicating FXR1’s high diagnostic efficacy in these cancer types, including CHOL, COAD, ESCA, HNSC, LIHC, LUAD, LUSC, PCPG, and STAD. Survival analysis disclosed a correlation between FXR1 expression and OS, PFI, and DSS in multiple tumor types. Overexpression of FXR1 was associated with poorer prognostic outcomes in most cancers, while it was linked to a better prognosis in GBM, LAML, and READ. These findings align with previous studies that underscored FXR1’s diagnostic value in BLCA and LUAD.^[14,17]^ The tumor immune microenvironment significantly influences tumor development. We investigated the relationship between FXR1 expression and the diverse molecular and immune subtypes across various cancer types, utilizing the TISIDB database. FXR1 expression exhibited variability within distinct cancer subtypes.

The most prevalent mutation events primarily involve amplifications, with FXR1 mutations frequently detected in LUAD, ESCA, OV, HNSC, and CESC. It is imperative to acknowledge that the initiation and progression of cancer are closely related with the accumulation of genetic variation.^[18,19]^Consequently, this research endeavors to elucidate alterations occurring within the FXR1 gene within a cohort of human cancer specimens. The FXR1 gene is situated at the chromosomal locus 3q26.3.^[4]^ Utilizing the cBioPortal Database, Diverse FXR1 Gene Alterations Emerge within the Cancer Cohort, with Prevalent Amplification Events in Pan-Cancer Cases, Implying Putative Oncogenic Roles for FXR1 Gene Mutations.

We constructed a PPI network to delve deeper into the biological functions of FXR1. This analysis revealed 10 central hub genes, establishing a strong correlation between their expression and FXR1 in various cancer types. This implies their synergistic roles in cancer biology. Notably, RNA-binding proteins (RBPs) play crucial roles in regulating post-transcriptional processes, encompassing RNA splicing, transportation, translation, and localization.^[20]^ Emerging research underscores the dysregulated expression and functionality of RBPs across various cancer types, including glioma, hepatocellular carcinoma, and colorectal cancer.^[21]^ Heterogeneous nuclear ribonucleoprotein K (hnRNPK) is a DNA/RNA-binding protein crucial in regulating various biological processes and disease development.^[22]^ It interacts with various nucleic acids and proteins, contributing to essential cellular functions such as transcription, translation, splicing, and chromatin remodeling.^[23]^ hnRNPK has been identified as an oncogene due to its elevated levels in different malignancies. Its oncogenic activity promotes cell proliferation, migration, and invasion.^[24–29]^ Tardna-binding protein (TARDBP), also known as TDP, is a highly conserved nuclear protein encoded by the TARDBP gene. It is expressed in multiple normal tissues throughout the human body.^[30]^ Studies have suggested that TARDBP may play a significant role in human tumorigenesis and has been detected in specific tumors like hepatocellular carcinoma and leukemia.^[31,32]^ Research has demonstrated that TARDBP acts as a regulator of glycolysis in hepatocellular cancer cells. It inhibits the expression of microRNA520, which subsequently affects the expression of platelet subtypes of phosphofructokinase, thereby modulating the glycolysis process in hepatocellular carcinoma.^[32]^ Additionally, TARDBP may facilitate the migration of lung cancer cells by regulating miR-423-3p. The decreased expression of miR-500a-3p has been associated with poor survival in lung cancer patients, suggesting that TARDBP may exert an inhibitory effect on cancer by regulating miR-500a-3p.^[33]^ NCBP2, also known as CBC2 (nuclear cap binding protein subunit 2), is a nuclear cap-binding protein complex component. Several cancers have been linked to this gene, including ovarian carcinoma, colon cancers, and lung cancers.^[34–36]^ In addition to inhibiting apoptosis, modulating signaling pathways, and responding to DNA damage, NCBP2 plays a critical role in cancer cell cycle regulation.^[34]^ XPO1 is the exclusive exporter of various proteins that suppress tumor growth and regulate the cell cycle. Research has demonstrated that in cancer, an overactive XPO1 leads to the abnormal export of crucial tumor suppressors such as p53, RB1, p21Cip1, and p27KIP1 to the cytoplasm, resulting in their functional inactivation.^[37,38]^ Overexpression of XPO1 has been observed in cancer patients and is associated with the progression of the disease, resistance to therapy, and shorter overall survival or progression-free survival.^[39]^ In addition to double-stranded DNA and RNA, DExH-Box helicase 9 (DHX9) unwinds other polynucleotide structures such as polyethylene glycol and cytidines.^[40]^ DHX9 serves as a key player within a spectrum of cellular activities, including regulating DNA replication, transcriptional regulation, RNA translation, translational machinery function, microRNA manipulation, circular RNA processing, and the safeguarding of genomic stability.^[41–43]^ Furthermore, DHX9 is a versatile enzyme with multiple domains and functions. When its regulation is disrupted, it can alter cellular growth and lead to the formation of tumors.^[42,44]^ Malignant tumors often exhibit abnormalities in mRNA translation, which contribute to their growth and metastasis.^[45]^ Current studies on dysregulated cancer translation primarily focus on the initiation of translation.^[46]^ This process is controlled by the eukaryotic translation factor 4F (EIF4F) heterotrimeric complex, consisting of EIF4A, EIF4E, and EIF4G.^[47]^ EIF4F facilitates the initiation of eukaryotic translation after the ribosome binds to the 5’ end of mRNA or the ribosome entry site (IRES) in its 5’ untranslated region.^[48]^ It is highly expressed in various cancers, including lung adenocarcinoma, lung squamous cell carcinoma, nasopharyngeal carcinoma, prostate cancer, breast cancer, and others.^[49–51]^ Karyopherin β1 (KPNB1), a member of the nuclear transport protein family, facilitates the transportation of protein molecules across the nuclear membrane Karyopherin β1 (KPNB1), belonging to the family of nuclear transport proteins, facilitates the transportation of protein molecules across the nuclear membranes.^[52]^ Scientific investigations have unveiled KPNB1’s implication in regulating cell proliferation in the context of lung cancer and head and neck cancer.^[53]^

Moreover, our enrichment analysis indicates that FXR1 could influence tumor etiology or pathogenesis via multiple pathways, encompassing cell cycle regulation, the electron transport chain oxphos system in mitochondria, complement and coagulation cascades, and the cornified envelope formation. Previous studies have found similar results. Notably, FXR1 is highly expressed in Wilms tumors and plays a crucial role in the original germ, marking a specific population of self-renewing progenitor cells in the fetal kidney.^[10]^ Moreover, there is evidence suggesting that depletion of FXR1 diminishes the ability of cells to induce migration of neighboring monocytes, potentially due to FXR1’s positive regulation of mRNA levels of specific cytokines and chemokines, such as IL1β and CCL2, which are involved in cell migration.^[54]^ Furthermore, studies have proposed that MRE11 and FXR1 may collaborate as cytoplasmic complexes to defend cells against mitochondrial reactive oxygen species, thereby reducing oxidative stress responses. Depleting FXR1 by siRNA increases sensitivity to mitochondrial reactive oxygen inducers, such as anthocyanins.^[55]^ In the context of hepatocellular carcinoma, FXR1 has been established as a factor that stimulates cell growth, movement, and infiltration by influencing the participation of SMAD2/3 in the TGF-β pathway.^[15]^ Additionally, FXR1 expression has been directly linked to the survival and proliferation of cancer cells, as it facilitates the cyclization of cMYC mRNA and promotes the recruitment of eukaryotic translation initiation factors to translation initiation sites, thereby elevating cMYC levels in cancer cells.^[4]^

Nowadays, an increasing number of studies have demonstrated the critical role of the immune microenvironment in the development and progression of tumors.^[56–58]^ In addition, our study discovered that immune cells’ expression level is correlated with cancer prognosis in various types. TIMER2 heat maps illustrated the relationship between FXR1 expression and macrophages, T cells CD4+, B cells, and T cells CD8 + infiltration. It was found that FXR1 expression was correlated with B cells, macrophages, T cells CD4+, and T cells CD8 + infiltration. A low and high expression group of FXR1 was created to determine the role that FXR1 plays on immune cells. As a result of these findings, we hypothesize that immune cells affect tumor survival in a manner that is correlated with the level of FXR1 expression. As a result of changes to gene expression, the immune microenvironment is altered, presenting a significant therapeutic potential for tumors.^[59,60]^ Further, we evaluated the correlation between the expression of FXR1 and the immune infiltration of cancer-associated fibroblasts in the TCGA across all cancer types. A correlation was found between FXR1 expression and immune infiltration by cancer-associated fibroblasts in specific tumors. It demonstrates the regulatory role of FXR1 in the immune microenvironment.

However, this is the first study to explore the multifaceted effect of FXR1 in human tumors, so it is imperative to acknowledge some limitations. Firstly, using multiple online databases introduces data heterogeneity, which may result in inconsistent findings. Secondly, since all results are based on observational studies, no causal conclusions can be drawn, and caution should be exercised to avoid over-interpreting the data. Some new analytical methods can also be selected to further analyze FXR1 molecules by statistical analysis of the relationship between FXR1 and other molecules, such as GCNCRF, DMFGAM, scRNA-seq, GCNAT, DCAMCP, NDALMA.^[61–67]^ Additionally, to validate these hypotheses, further in vitro and in vivo experiments are needed to validate the hypothesis that FXR1 may be a diagnostic/prognostic biomarker for certain cancers and a target for immunotherapy. Lastly, extensive clinical sample data collection and analysis is necessary to fully utilize FXR1 as a predictive biomarker for different cancers. This should involve the establishment and refinement of inclusion criteria, considering various clinicopathological parameters such as sex, age, pathological classification, and tumor differentiation while accounting for factors that contribute to tumor heterogeneity and individual differences. Molecular studies addressing these questions will be crucial to future research endeavors.

## 5. Conclusions

In summary, this research comprehensively analyzed FXR1 across various types of cancer, revealing significant correlations between FXR1 expression and clinical prognosis, mutation, and immune cell infiltration. This investigation encompassed a comprehensive analysis of FXR1 across a spectrum of cancer subtypes, uncovering significant correlations between FXR1 expression and clinical prognostic outcomes, mutational profiles, and the presence of immune cell infiltrations. The study also shed light on the potential role of FXR1 as a prognostic indicator.

## Author contributions

**Conceptualization:** Xinghua Li.

**Data curation:** Keyuan Xiao, Ihsan Ullah, Fan Yang, Xinghua Li.

**Funding acquisition:** Keyuan Xiao.

**Visualization:** Xinghua Li.

**Writing – original draft:** Keyuan Xiao.

**Writing – review & editing:** Ihsan Ullah, Fan Yang, Jiao Wang, Chunxia Hou, Yuqiang Liu, Xinghua Li.
